# Increased Growth Differentiation Factor 15 Levels Are Associated with HIV-Associated Neurocognitive Impairment: A Pilot Study

**DOI:** 10.3390/brainsci15010049

**Published:** 2025-01-07

**Authors:** Ali Boustani, Mary K. Ford, Jacqueline R. Kulbe, Anna E. Laird, Leeann Shu, Matthew Spencer, Bryant Avalos, Kyle C. Walter, Ronald J. Ellis, Jerel Adam Fields

**Affiliations:** 1 Department of Psychiatry, University of California San Diego, San Diego, CA 92093, USA; aboustani@health.ucsd.edu (A.B.); mkford@health.ucsd.edu (M.K.F.); jkulbe@health.ucsd.edu (J.R.K.); alaird@health.ucsd.edu (A.E.L.); l1shu@ucsd.edu (L.S.); m3spencer@health.ucsd.edu (M.S.); bavalosleyva@health.ucsd.edu (B.A.); kcwalter24@gmail.com (K.C.W.); 2 Department of Neurosciences, University of California San Diego, San Diego, CA 92093, USA; roellis@health.ucsd.edu

**Keywords:** GDF15, HIV-associated NCI, neuroinflammation

## Abstract

**Background/Objectives**: HIV-associated neurocognitive impairment (NCI) remains a prevalent issue among people with HIV (PWH) despite advancements in antiretroviral therapy (ART). The pathogenesis of HIV-associated NCI is linked to chronic neuroinflammation caused by HIV, even in those with successful viral suppression. Growth Differentiation Factor 15 (GDF15), a protein involved in inflammatory and metabolic stress responses, has emerged as a key player and potential biomarker for various neurological conditions. This study investigates the relationship between GDF15 expression and HIV-associated NCI. **Methods**: PWH from the California NeuroAIDS Tissue Network (CNTN) underwent comprehensive neuropsychological exams within 12 months before death and were categorized based on cognitive performance. We examined GDF15 levels in their CSF (Cerebrospinal Fluid) and brain tissues using immunoblotting, immunohistochemistry, double immunolabeling, and ELISA. **Results**: The cohort was of a similar age across HIV-associated NCI statuses (mean = 40.5), with a predominance of males (77%). The mean plasma viral load was 3.56 log_10_ copies/mL for Neurocognitively Unimpaired (NUI) PWH and 5.38 log10 copies/mL for people with HIV-associated NCI. GDF15 protein levels were significantly elevated in the frontal cortices of PWH with NCI compared to NUI PWH. **Conclusions**: The findings indicate that GDF15 may play a role in the pathogenesis of HIV-associated NCI, possibly through neuroinflammatory mechanisms. The strong association between GDF15 levels and cognitive impairment severity suggests its potential as a biomarker for the early detection and monitoring of NCI in PWH.

## 1. Introduction

Despite significant advances in antiretroviral therapy (ART), HIV-associated neurocognitive impairment (NCI) continues to pose a challenge in the management of people with HIV (PWH) [1,2]. The etiology of HIV-associated NCI remains under investigation. Previous studies report that HIV can cause chronic inflammation in the brain despite ART, which can lead to neuronal damage and degeneration over time [3,4]. This inflammation can be caused by both HIV’s immune response and ART, which disrupts the balance of chemokines in the cerebrospinal fluid (CSF) and plasma [5]. This chemokine disruption can cause metabolic dysfunction [6], which, in turn, contributes to the pathogenesis of HIV-associated NCI [7].

With the advent and improvement of ART, the landscape of neurocognitive impairment in PWH has shifted significantly. The most severe form of cognitive decline, HIV-Associated Dementia (HAD), has become increasingly rare [8], leading to a new classification that simplifies the categorization of PWH into two groups: NUI PWH and those with HIV-associated NCI [9]. Although the current classification of PWH divides individuals into NUI PWH and those with HIV-associated NCI, this approach may overlook the spectrum of cognitive impairment seen in previous categorizations, such as the established HIV-associated neurocognitive disorder (HAND) framework. The HAND framework categorizes PWH into three distinct groups: asymptomatic neurocognitive impairment (ANI); mild neurocognitive disorder (MND); and HAD [10]. To ensure a comprehensive analysis, we aim to incorporate both the newer binary classification and the former HAND grouping in this investigation. Through this comprehensive approach, we aim to identify Growth Differentiation Factor 15 (GDF15) expression patterns for varying brain cell types, HIV-associated stimuli, and interventional strategies, with the ultimate goal of identifying novel therapeutic targets for the prevention, identification, and management of HIV-associated NCI.

Despite the critical need for early detection of HIV-associated NCI, current diagnostic approaches rely heavily on neurocognitive testing, which can be time-consuming and may miss early changes. While several biomarkers have been investigated, including neurofilament light chain (NFL), S100B, and inflammatory cytokines, none have demonstrated sufficient sensitivity and specificity for clinical implementation [11,12].

GDF15 emerged as a promising candidate due to its unique position at the intersection of inflammatory and metabolic pathways, which are dysregulated in HIV infection [13,14]. Previous studies have shown GDF15 elevation in various neurological conditions characterized by inflammation, including Alzheimer’s disease and multiple sclerosis, suggesting its potential utility in HIV-associated NCI [15,16,17]. Moreover, GDF15’s role in cellular stress responses and tissue adaptation makes it particularly relevant to the chronic inflammatory state observed in PWH. Our previous works in postmortem brain specimens from PWH illustrate alterations in metabolic pathways, including mitochondrial dynamics, biogenesis, and autophagic flux [18,19]. Thus, there may be a role for GDF15 in modulating these maladaptive responses to chronic HIV infection in the brain. Increasing data regarding GDF15’s role in stress introduce GDF15 as a hormone conveying somatic distress to the brain [20].

The immune system depends on a delicate balance between proinflammatory and anti-inflammatory cytokines to effectively respond to pathogens while preventing excessive tissue damage and maintaining tissue homeostasis [21,22]. Proinflammatory pathways are activated in response to infection, injury, or other tissue damage to eliminate pathogens and clear debris [23]. On the other hand, anti-inflammatory pathways act as a counterbalance to proinflammatory pathways for dampening excessive inflammation, resolving immune responses, and promoting tissue repair and regeneration [24]. Anti-inflammatory responses are mediated by various mechanisms, including the production of anti-inflammatory cytokines such as interleukin-10 (IL-10) and transforming growth factor-beta (TGF-β) [22]. These cytokines inhibit the production of proinflammatory cytokines and promote the differentiation of regulatory immune cells, such as M2 macrophages, which help suppress immune responses and promote tissue repair and adaptation to chronic inflammation [25,26,27,28].

GDF15 has recently attracted considerable interest as a biomarker due to its anti-inflammatory and metabolic roles in facilitating tissue adaptation in response to stressors [29,30]. GDF15 is often considered a biomarker of cellular stress and injury as its levels tend to increase in response to various insults to the body, such as infection, inflammation, neoplasms, or tissue damage [31,32,33,34]. It is believed to exert its effects through binding to specific receptors on cells, leading to various downstream signaling pathways that regulate processes like cell survival, metabolism, and immune responses [29,33]. For instance, GDF15 binds to its receptor, called GDNF Family Receptor Alpha-Like (GFRAL), which is primarily expressed in the area postrema (AP) and nucleus of the solitary tract (NTS) in the brainstem. These regions are involved in the regulation of appetite and body weight [35,36,37,38,39].

The aim of the study is to investigate GDF15 protein expression in brains and CSF of PWH with HIV-Associated NCI, including the pattern of GDF15 expression in microglia, neurons, and astrocytes to test the hypothesis that there is an association between the pattern and abundance of GDF15 expression and HIV-Associated NCI.

## 2. Materials and Methods

### 2.1. Study Population

For the present study, we included 2 cohorts of individuals diagnosed with HIV (22 participants for the brain cohort and 29 participants for the CSF cohort), stratified into categories of NUI or HIV-associated NCI [Table 1], from the California Neuro-Acquired Immune Deficiency Syndrome (AIDS) Tissue Network (CNTN) (Institutional Review Board [IRB] # 080323) at the University of California, San Diego. We scoped 10 years of autopsy dates between 1999 and 2009 since the PWH with advanced stages of HIV-associated NCI were more abundant in the early 2000s, so thus helped us compare various stages of HIV-associated NCI more comprehensively. Cases had neuromedical and neuropsychological examinations within a median of 12 months before death. Subjects were excluded if they had a history of CNS opportunistic infections or non-HIV-related developmental, neurologic, psychiatric, or metabolic conditions that might affect CNS functioning (e.g., loss of consciousness exceeding 30 min, psychosis, substance dependence). HIV-associated NCI diagnoses were determined from a comprehensive neuropsychological test battery administered according to standardized protocols [40]. Study participants underwent standardized neuropsychological testing assessing seven cognitive domains: verbal fluency; executive function; information processing speed; learning; recall; working memory; and motor function. Raw scores were converted to demographically adjusted T-scores using published normative data [41]. HIV-associated NCI was diagnosed based on performance at least 1 standard deviation below the mean in at least two cognitive domains, following established criteria [42]. Their CSF samples were collected through lumbar puncture during their visits. Most cases died as a result of acute bronchopneumonia or septicemia, and brain tissues were collected with autopsy within 24 h of death (median postmortem interval was 12 h).

### 2.2. Immunoblotting

Frontal cortex tissues (0.1 g) from Brodmann areas 46 and 9 were sonicated in a fractionation buffer containing a complete protease inhibitor cocktail (Roche, Basel, Switzerland, cat. No. 04693116001). Samples were precleared via centrifugation, and the supernatant was retained as the whole lysate. After determination of the protein content of all samples by BCA Protein assay (Thermo Fisher Scientific, Waltham, MA, USA, Cat. no. 23225), samples were denatured in Laemmli Sample buffer (Bio-Rad, Cat. no. 1610747) and 2-Mercaptoethanol (Bio-Rad, Hercules, CA, USA, Cat. no. 1610710). Samples were loaded 15 μg per well onto 4–15% Criterion TGX stain-free gels (Bio-Rad, Cat. no. 5678085). Then, samples were electrophoresed in Tris/Glycine/SDS running buffer (Bio-Rad, Cat. no. 1610772). Proteins were transferred onto an LF PVDF membrane (Bio-Rad, Cat. no. L002048) with transfer stacks (Bio-Rad, Cat. no. L002044 B) and transfer buffer (Bio-Rad, Cat. no. 10026938) using a Bio-Rad Trans-Blot Turbo transfer system. After the transfer, total protein was imaged using a Bio-Rad ChemiDoc imager under the stain-free blot setting to control the transfer quality. The membranes were then blocked with 1× Tris Buffered Saline with 1% Casein (Bio-Rad, Cat. no: 1610782) for one hour before being incubated overnight at 4 °C with the primary antibody, mouse anti-GDF15 (Invitrogen, Waltham, MA, USA, cat. no. ma5-31346; 1:1000 in blocking buffer) and *β*-actin (Abcam, Cambridge, UK, Cat. no. ab6276; 1:1000 in blocking buffer). Anti-mouse IgG, HRP-linked antibody was used as the secondary antibody for 1 h at room temperature (Bio-Rad, cat. no. 170-6516; 1:5000 in PBS-T), visualized with SuperSignal West Femto Maximum Sensitivity Substrate (ThermoFisher Scientific, Cat. no. 34095). Images were obtained, and semi-quantitative analysis was performed with the ChemiDoc gel imaging system and Image Lab Software (Bio-Rad version 6.1). The band density was quantified and normalized to *β*-actin and graphed with the Prism software (version 10.2.3).

### 2.3. Immunohistochemistry

Paraffin-embedded brain tissues from Brodmann areas 46 and 9 in the frontal cortex were dewaxed in histoclear (National Diagnostics, Atlanta, GA, USA, cat. no. HS-200), rehydrated in a decreasing graded ethanol series, and heated at 90–95 °C in citrate buffer (Sigma Aldrich, St. Louis, MO, USA, cat. no. C9999) for antigen retrieval. Tissues were pretreated for 20 min with 1% H_2_O_2_ and incubated at 4 C overnight with the primary antibody, GDF15 (Invitrogen, ma5-31346), diluted 1:400 in DAKO CAD (Agilent Technologies, St. Clara, CA, USA, cat. no. S3022). Sections were then incubated in secondary antibody diluted 1:100 in PBS, Impress HRP Anti-mouse IgG (Vector, Stuttgart, Germany, cat. no. MP-7402) for 40 min at room temperature, followed by NovaRED peroxidase (HRP) substrate made with NovaRED Peroxidase (HRP) Substrate Kit as per manufacturer’s instructions (Vector, cat. no. SK-4805). Samples were then stained in Hematoxylin (1:5; Ricca, Arlington, TX, USA, cat. no. 3530-32). Control experiments consisted of incubation with secondary antibody only to determine if any staining observed was due to non-specific binding rather than specific interaction with the target antigen. The tissues were left to dry at 60 C for an hour and coverslipped with cytoseal 60 (Thermo Scientific, cat. no. 8310-16). Immunolabeled sections were scanned with the Image-Pro Plus program (Media Cybernetics, Silver Spring, MD, USA). For each case, a total of four images were analyzed to estimate the average intensity of the immunolabeling.

### 2.4. Double-Immunolabeling

Double-immunolabeling of tissues was performed to elucidate the spatial distribution and potential colocalization of specific antigens within cellular compartments. Tissue was processed and pre-treated as above and sections were incubated with the following combination of primary antibodies: GDF15 (Invitrogen, MA5-31346, diluted 1:500 in PBS (Phosphate-buffered saline)) and GFAP (Dako, cat. no. Z0334, diluted 1:500 in PBS); GDF15 and MAP2 (BioLegend, San Diego, CA, USA, cat. no. 840601, diluted 1:1000 in PBS) t; and GDF15 and IBA1 (Fujifilm Wako’s, Osaka, Japan, cat. no. 019-19741, diluted 1:1000 in PBS). Then, it was followed by secondary antibodies conjugated to fluorophores with distinct emission spectra. These secondary antibodies were anti-mouse (Fluorescein, cat. no. FI-2000, diluted 1:100 in PBS, and emission of 515 nm), used to detect GDF15, and anti-rabbit (Invitrogen, cat. no. A21245, diluted 1:100 in PBS, and emission of 647 nm), used to detect GFAP, MAP2, and IBA1. Double-immunolabeled sections were imaged with a Zeiss 880 Airyscan Confocal microscope, and images were captured and saved with ZEN 2.3 SP1 software. Colocalization analysis of fluorescence microscopy images was conducted using the JACoP (Just Another Colocalization Plugin) plugin (v2.1.4) for Fiji software (2.14.0/1.54f). By computing Manders split coefficients (M1 and M2), we accurately quantified and characterized the degree of colocalization between GDF15 and various cellular markers in fluorescence microscopy images [43].

### 2.5. Enzyme-Linked Immunosorbent Assay (ELISA)

For this assay, CSF from PWH and the supernatants of the treated cell were used to run with the Human GDF-15 ELISA Kit from Raybiotech (cat. no. ELH-GDF15, Peachtree Corners, GA, USA). This assay uses an antibody specific to Human GDF15 coated on a 96-well plate. A total of 100 µL of standards and CSF samples from PWH were pipetted into the wells, and the plate was incubated at 4 °C overnight. The wells were washed, and a biotinylated anti-human GDF15 antibody was added. After washing, samples were treated with HRP-conjugated streptavidin followed by washing, addition of TMB substrate, and stop solution. Samples were evaluated in BioTek Synergy HTX Multimode Reader and analyzed with Gen5 software (version 3.03) for optical density at 450 nm per the manufacturer’s instruction.

### 2.6. Statistical Analysis

Data were analyzed with the Prism software (version 10.2.2). Comparisons among groups were performed with one-way ANOVA with the post hoc Tucky’s test and unpaired Student’s *t*-test where appropriate. All results were expressed as mean ± SEM. The differences were considered to be significant if *p*-values were <0.05.

We have used Cohen’s *d* to quantify the magnitude of differences between groups [44], allowing us to interpret the practical significance of our findings. Cohen’s *d* provides a standardized measure of the effect size, offering a refined interpretation beyond statistical significance alone. This is especially important when investigating subtle cognitive differences in people with HIV as it helps us determine whether the observed group differences in neurocognitive impairment are clinically meaningful. We interpret the effect sizes using the conventional thresholds proposed by Cohen, where 0.2 indicates a small effect, 0.5 is a medium effect, and 0.8 is a large effect. For HIV-associated NCI, power calculations using preliminary data indicated that participants would provide 80% power to detect a moderate effect size (Cohen’s *d* = 0.6) in GDF15 levels between groups at α = 0.05.

## 3. Results

### 3.1. Clinical Characteristics of the Human Cohort

In this study, participants were assessed for NCI, sex, age, ART usage, plasma viral load (log copies/mL), and CD4 count between 1999 and 2009. The cohorts were of similar age across HIV-associated NCI statuses, with a predominance of males (77% in the Brain cohort and 82% in the CSF cohort) [Table 1]. NUI members of the brain cohort had lower plasma viral loads than those with HIV-associated NCI but higher CD4 counts, while there was no significant difference in the CSF cohort [Table 1].

### 3.2. GDF15 Is Increased in the Frontal Cortex Lysates from PWH with HAD

**Figure 1:**
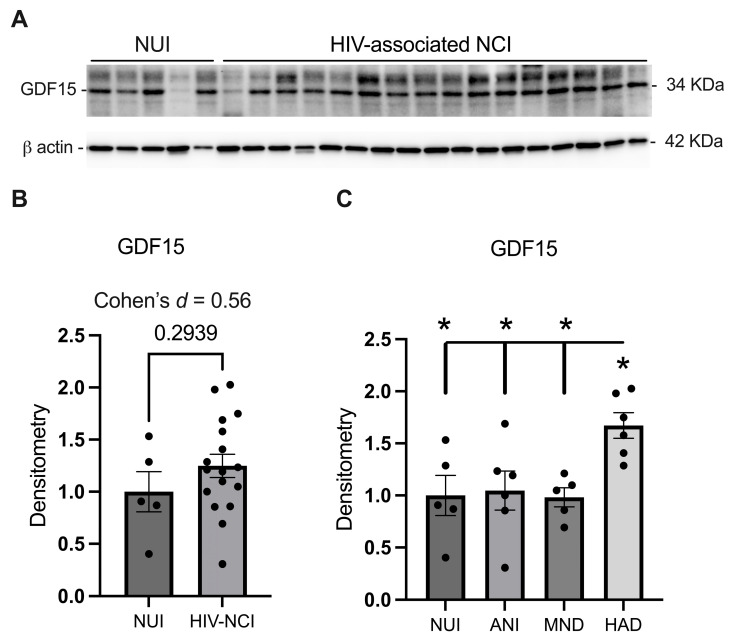
GDF15 is increased in the frontal cortex lysates from PWH with HAD. (**A**) Immunoblot for mature GDF15 and *β* actin from PWH. (**B**) Band densitometry quantification of GDF15 comparing GDF15 expression in NUI PWH and those with HIV-associated NCI. (**C**) Band densitometry quantification of GDF15 in all varieties of HAND. Data were analyzed via (**B**) student’s *t*-test and effect size and (**C**) one-way ANOVA; * *p* < 0.05. Mean ± SEM. GDF15 = growth differentiation factor 15; PWH = people with HIV; NUI = neurocognitively unimpaired; NCI = neurocognitive impairment; ANI = asymptomatic neurocognitive impairment; MND = mild neurocognitive disorders; HAD = HIV-associated dementia.

Immunoblotting of the mature GDF15 protein isoform with a mass of 34 kDa was performed on the frontal cortex total lysates from NUI PWH and those with HIV-associated NCI [Figure 1A]. GDF15 expression does not significantly increase in the frontal brain lysates of PWH with HIV-associated NCI. However, effect size analysis (Cohen’s *d* = 0.56) suggests a moderate difference, which may have potential clinical relevance [Figure 1B]. One-way ANOVA revealed a statistically significant difference in GDF15 expression between HAD and other groups (*p* < 0.05), with GDF15 expression being increased approximately two-fold compared to non-HAD cases [Figure 1C].

### 3.3. GDF15 Is Increased in Gray Matter in the Frontal Cortices of Brain Specimens from PWH with HIV-Associated NCI

**Figure 2:**
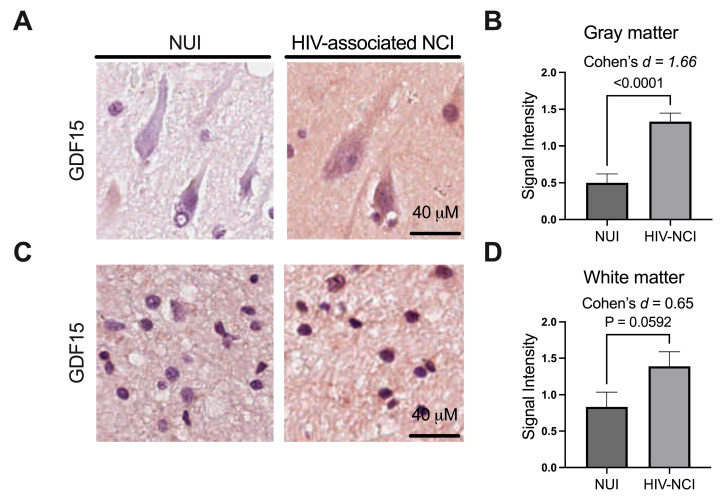
GDF15 is increased in the gray matter in the frontal cortices of brain specimens from PWH with HIV-associated NCI. (**A**) Representative image of paraffin-embedded frontal cortex sections from NUI PWH and those with HIV-associated NCI stained for GDF15 in the gray matter and (**B**) quantification of grey matter GDF15 intensity. (**C**) Representative image of GDF15 staining in white matter and (**D**) quantification of white matter GDF15 intensity. Mean ± SEM. Statistical analysis by *t*-test and effect size analysis. NUI = neurocognitively unimpaired; NCI = neurocognitive impairment; GDF15 = growth differentiation factor 15.

To determine where GDF15 expression is localized in the frontal cortex, we performed immunohistochemistry on paraffin-embedded frontal cortex sections from NUI and HIV-associated NCI cases and analyzed GDF15 signals in the grey and white matter. We stained 18 slides for each group and analyzed the slides by blinded scoring. GDF15 signal was increased in the gray matter of HAND cases compared to non-HAND cases [*p* < 0.0001, Figure 2A,B]. Similarly, a less robust increase in GDF15 signal intensity was observed in the white matter of HAND frontal cortices compared to non-HAND cases [*p* = 0.0592; Figure 2C,D].

### 3.4. GDF15 Expression Is Increased in Microglia and Neurons in PWH with HIV-Associated NCI

One of our main objectives was to evaluate GDF15 expression within different brain cell types across varying HAND severities. Therefore, frontal cortex sections from four participants in each group were stained with GDF15 plus IBA1 (microglia), MAP2 (neurons), or GFAP (astrocytes). Subsequently, confocal microscope images were captured twice per slide and analyzed for signal colocalization.

**Figure 3:**
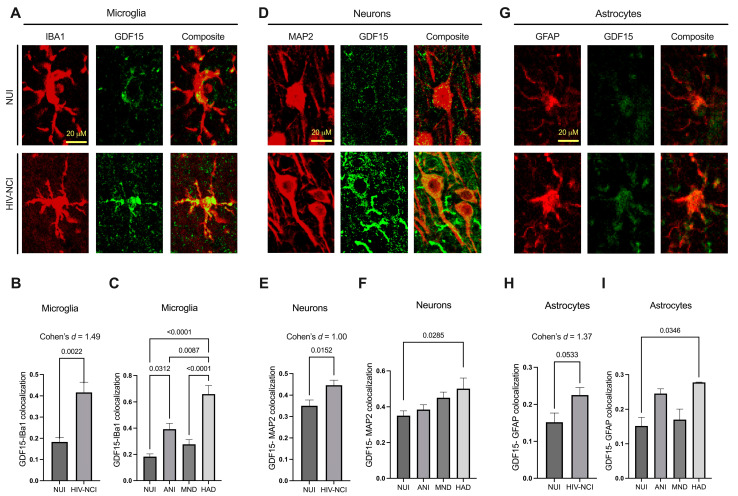
GDF15 expression is increased in microglia and neurons in PWH with HIV-associated NCI. (**A**) Representative confocal microscope image of IBA1 and GDF15 colocalization. (**B**) Percentage of GDF15 signal colocalized with IBA1 signal in NUI PWH and those with HIV-associated NCI and (**C**) in varying severities of HAND. (**D**) Representative confocal microscope image of MAP2 and GDF15 colocalization. (**E**) Percentage of GDF15 signal colocalized with MAP2 signal in NUI PWH and those with HIV-associated NCI and (**F**) in varying severities of HAND. (**G**) Representative confocal microscope image of GFAP and GDF15 colocalization. (**H**) Percentage of GDF15 signal colocalized with GFAP signal in NUI PWH and those with HIV-associated NCI and (**I**) in varying severities of HAND. (**B**,**E**,**H**) were analyzed via *t*-test and effect size. (**C**,**F**,**I**) were analyzed via one-way ANOVA with post hoc Tukey’s test. Mean ± SEM. GDF15 = growth differentiation factor 15; PWH = people with HIV; NUI = neurocognitively unimpaired; NCI = neurocognitive impairment; HAND = HIV-associated neurocognitive disorder. ANI = asymptomatic neurocognitive impairment; MND = mild neurocognitive disorder; HAD = HIV-associated dementia.

There was a statistically significant difference in GDF15-IBA1 and GDF15-MAP2 co-localization between NUI PWH and those with HIV-associated NCI [Figure 3B,E]. Post hoc analysis by Tukey revealed that the most robust increases in GDF15 expression occurred in microglia, in which GDF15 was significantly increased in both ANI (*p* = 0.03) and HAD (*p* < 0.0001) cases compared to NUI PWH [Figure 3C]. However, GDF15 in neurons (*p* = 0.0285) and astrocytes (*p* = 0.0346) was also significantly increased in HAD cases compared to NUI PWH [Figure 3F,I].

### 3.5. CSF GDF15 Levels Do Not Show a Statistically Significant Increase in HIV-Associated NCI; However, the Effect Size Analysis Suggests a Moderate Increase

**Figure 4:**
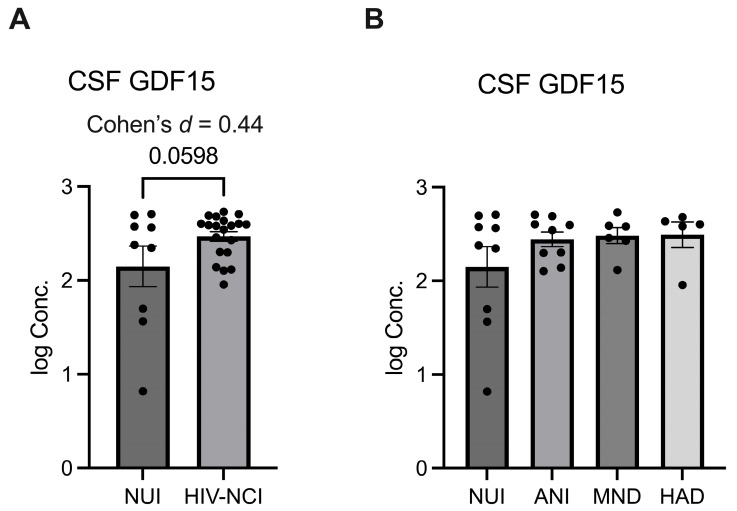
CSF GDF15 levels do not show a statistically significant increase in HIV-associated NCI; however, the effect size analysis suggests a moderate increase. (**A**) Log transformed GDF15 concentration (pg/mL) as measured by ELISA for CSF specimens from NUI PWH and those with HIV-associated NCI and in (**B**) NUI PWH and varying severities of HAND. (**A**) was analyzed via *t*-test and effect size, and (**B**) was analyzed via one-way ANOVA. Mean ± SEM. GDF15 = growth differentiation factor 15; CSF = cerebrospinal fluid; NUI = neurocognitively unimpaired; NCI = neurocognitive impairment; HAND = HIV-associated neurocognitive disorder; PWH = people with HIV; ANI = asymptomatic neurocognitive impairment; MND = minor neurocognitive disorder; HAD = HIV-associated dementia.

ELISA was used to quantify levels of GDF15 in CSF samples from NUI PWH and those with varying stages of HAND (ANI, MND, HAD). Data were log-transformed. One-way ANOVA revealed that there was not a significant difference across groups. However, the *t*-test revealed a near significant (*p* = 0.0598) increase in CSF GDF15 when all HAND severities were combined and compared to NUI cases, aligning with increases in tissue expression of GDF15 in HAND [Figure 4].

The observed trend toward increased CSF GDF15 in HIV-associated NCI (*p* = 0.0598) suggests potential utility as a biomarker, particularly when combined with other measures. Effect size analysis (Cohen’s *d* = 0.44) indicates a moderate trend to increase, supporting further investigation in larger cohorts.

## 4. Discussion

The findings of this study indicate, for the first time, an association between increased levels of GDF15 and the severity of HIV-associated NCI in PWH. The data demonstrate that GDF15 expression is elevated in the frontal cortex and CSF of PWH, particularly in those with more severe forms of HAND, such as HAD. Furthermore, this study provides novel insights into the cellular sources of GDF15 within the brain, showing its increased expression in microglia, neurons, and astrocytes, with the most pronounced elevation observed in microglia.

GDF15 is increasingly recognized for its immunomodulatory role in various physiological and pathological conditions [45]. It is upregulated in response to cellular stressors, including inflammation, hypoxia, and metabolic disturbances, and controls immune responses [46]. Studies have shown that GDF15 can suppress the production of proinflammatory cytokines, inhibit T-cell activation, and modulate macrophage polarization, favoring an anti-inflammatory phenotype [47,48]. Additionally, its ability to regulate immune cell migration and attenuate chronic immune activation suggests a broader role in limiting tissue damage during sustained inflammation [49]. These properties position GDF15 as a key mediator of immune homeostasis.

On the other hand, substantial data have shown that neoplastic cells can escape both innate and adaptive immune responses utilizing this immunomodulatory role of GDF15 [34,50,51,52]. Similarly, virally infected cells may survive the immune response through this pathway [53]. It has been reported that placental GDF15 protects the fetus against maternal immune response to fetal antigens by regulating immune cell function [54,55,56].

GDF15’s anti-inflammatory and tissue-adaptive properties suggest that its upregulation may be a compensatory response to the chronic neuroinflammation and cellular stress induced by HIV infection and its treatment with ART. Previous research has highlighted the persistent inflammation in the brains of PWH despite ART, which can lead to neuronal damage and degeneration over time [3].

Previous studies have found that GDF15 is localized to microglia, neurons, and astrocytes in the brain, where it is particularly enriched in microglial cells under pathological conditions [50,57,58]. In peripheral tissues, GDF15 is expressed in macrophages, adipocytes, and epithelial cells, with its levels significantly increasing in response to stress signals such as hypoxia, inflammation, and metabolic disturbances [16,59]. These findings highlight the cell-specific expression of GDF15 across both central and peripheral systems, suggesting a critical role in coordinating stress and immune responses in diverse tissue environments.

These data are consistent with our findings that GDF15 is localized in microglia, neurons, and astrocytes, which is particularly noteworthy, as these cell types play critical roles in the brain’s response to injury and inflammation. Microglia, the brain’s resident immune cells, are key mediators of neuroinflammation. The significant increase in GDF15 expression in microglia, especially in HAD cases, suggests that GDF15 may be involved in modulating microglial activation and the inflammatory response in HIV-associated NCI. Similarly, the elevated GDF15 levels in neurons and astrocytes, even though to a lesser extent, indicate that these cells may also contribute to the observed neuroinflammatory conditions in the brains of PWH with HIV-associated NCI.

Careful consideration of potential systemic effects would be needed given GDF15’s diverse physiological roles. Integration of GDF15 measurement with current clinical assessments could enhance early detection of HIV-associated NCI. Cost-effectiveness studies comparing GDF15 testing to current diagnostic approaches would help establish its clinical utility. Additionally, an investigation of how GDF15 levels relate to other established biomarkers could lead to the development of more accurate diagnostic panels.

Future studies should address several key questions raised by our findings. First, longitudinal studies tracking GDF15 levels from early HIV infection through the development of cognitive symptoms could establish its utility as a predictive biomarker. Second, investigation of sex-specific effects is crucial, given known differences in inflammatory responses between males and females. Third, mechanistic studies exploring GDF15 signaling pathways in brain cells could identify therapeutic targets. Fourth, evaluation of GDF15 in more accessible biospecimens (e.g., plasma) could facilitate clinical translation. Last but not least, in vitro studies could confirm the findings of our study by exposing the brain microenvironment to various HIV-related stimuli such as ART and inflammatory and metabolic mediators.

While our study provides valuable insights into the role of GDF15 in HIV-associated NCI pathogenesis, several limitations should be considered when interpreting the results. First, this study did not specifically account for sex differences, which could influence the observed outcomes given the known sexual dimorphism in immune responses and neuroinflammatory processes. Additionally, the relatively small number of PWH diagnosed with HAD, particularly after ART development, limits the generalizability of our findings and precludes robust subgroup analyses. This limitation underscores the need for larger cohort studies encompassing diverse populations to validate our observations and elucidate the relation between GDF15 dysregulation, HIV infection, and neurocognitive impairment. What is more, we included a cohort of PWH with more advanced HIV-associated NCI to be able to study the changes in GDF15 across the HIV-associated NCI. However, these participants belong to the pre-ART era, when most of them were not virally suppressed, which may not reflect the current clinical landscape, where most PWH have suppressed plasma viral loads. The cross-sectional nature of our study precludes the determination of whether GDF15 elevation precedes or follows cognitive decline. While we controlled for major confounding conditions, the impact of different ART regimens on GDF15 expression requires further investigation. Post-mortem changes may affect protein expression, although our short post-mortem interval (median 12 h) minimizes this concern.

## 5. Conclusions

In conclusion, this study highlights the association between GDF15 levels and HIV-associated NCI severity in PWH, suggesting a role for GDF15 in the pathophysiology of HIV-associated NCI. The upregulation of GDF15 in microglia, neurons, and astrocytes underscores its potential involvement in the brain’s response to HIV-induced neuroinflammation. These findings pave the way for further research into GDF15 as a biomarker and therapeutic target for HIV-associated NCI, with the ultimate goal of improving the management and quality of life of PWH.

## Figures and Tables

**Table 1:** Clinical characteristics of the human cohorts. NCI: neurocognitive impairment; ART: antiretroviral therapy; HAND: HIV-associated neurocognitive disorder; ANI: asymptomatic neurocognitive impairment; MND: mild neurocognitive disorder; HAD: HIV-associated dementia.

			Sex (f/m)	Age	ART Duration (Months)	Plasma Viral Load	CD4
Brain cohort	NUI		0/5	44.4 ± 7.6	11 ± 16.3	3.5 ± 0.6	196.2 ± 156.9
	HIV-associated NCI		4/12	39.5 ± 4.4	7.4 ± 10.6	5.3 ± 0.8	40.3 ± 30.4
	HAND	ANI	2/3	37 ± 6.7	4.1 ± 4.1	5.3 ± 0.8	29.8 ± 32.8
		MND	1/4	35.6 ± 8.9	13.4 ± 17.3	5.7 ± 0.9	7.2 ± 5.9
		HAD	1/5	45.2 ± 8.2	4.6 ± 6.7	5.4 ± 0.8	78.4 ± 115.9
CSF cohort	NUI		1/8	43.7 ± 7.4	4.3 ± 4.1	5.6 ± 1.7	54.2 ± 102.1
	HIV-associated NCI		4/16	43.7 ± 9.8	5.9 ± 6.4	5.5 ± 1.6	146.5 ± 243.7
	HAND	ANI	2/7	44.5 ± 13.4	6.3 ± 7.3	5.0 ± 1.5	153.6 ± 216.8
		MND	1/5	42.6 ± 3.8	6.6 ± 5.5	5.7 ± 0.9	183.6 ± 349.1
		HAD	1/4	43.6 ± 9.0	4.2 ± 6.7	5.7 ± 1.8	75.0 ± 134.7

## Data Availability

The raw data supporting the conclusions of this article will be made available by the authors on request.
